# Effect of Reinforced Self-Cured Acrylic Resin on Flexural Strength

**DOI:** 10.1155/2022/2698995

**Published:** 2022-08-24

**Authors:** Chavinee Apimanchindakul, Pheeradej Na Nan, Napapa Aimjirakul

**Affiliations:** ^1^Department of Conservative Dentistry and Prosthodontics, Srinakharinwirot University, Bangkok, Thailand; ^2^Ministry of Digital Economy and Society, Bangkok, Thailand

## Abstract

**Introduction:**

The aim of this study was to determine the effect on the flexural strength of the self-cured acrylic resin by incorporating short E-glass fiber (SEGF) and ultra-high-molecular-weight polyethylene (UHMW-PE) filler in the acrylic resin.

**Methods:**

Fifty-six rectangular (64 × 10 × 3.3 mm^3^) (ISO standard 20795–1:2013) self-cured acrylic resin specimens were fabricated and divided into seven groups per test, according to the percent by weight of SEGF and UHMW-PE filler (*n* = 8). Each testing group entails a control group and an addition of 1% and 2% SEGF, 1% and 2% UHMW-PE, 0.5% SEGF/UHMW-PE, and 1% SEGF/UHMW-PE. A three-point bending test was conducted to obtain the flexural strength of each specimen. The fractured surfaces of the specimens were evaluated, and a scanning electron microscope view was taken. Test results were statistically analyzed with one-way ANOVA and Tukey HSD tests (*p* value<0.05).

**Results:**

The flexural strength of self-cured acrylic resin with the addition of 1% SEGF and 1% SEGF/UHMW-PE (50.93, 46.13 MPa) was significantly higher than that of the control group (41.72 MPa) (*p* < 0.05). Nonetheless, the addition of 1% UHMW-PE (39.34 MPa) showed the lowest flexural strength, significantly lower than other experimental groups, except the control group.

**Conclusion:**

The addition of 1% SEGF significantly improves the flexural strength of the self-cured acrylic resin denture base.

## 1. Introduction

The survival and success of denture prosthesis is significantly influenced by the choice of appropriate materials. The fabrication of dentures in achieving a natural appearance and the ability to withstand the harsh oral environments and tremendous masticatory forces is still a challenge for clinicians. For the past eighty years, the poly (methyl) methacrylate (PMMA) resin remains the main constitute for denture base construction. Dentures' fractures are common and cause much distress and cost for patients and dentists. Ideal denture repair material should have adequate flexural strength as it plays a crucial role in how well the resin will perform under masticatory stress and to resist denture deformation during function. Most fractured dentures are repaired using self-cured or autopolymerized acrylic resin, in which it results in the insufficient transverse strength of approximately only 60% to 65% of the original strength [1]. However, it is a simple, quick, and inexpensive procedure and can be performed chairside with a faster turnover of lab work. Even though self-cured acrylic resin is still far from ideal in meeting all ideal physical, mechanical, chemical, and biological properties, it has insufficient transverse strength compared to conventional heat-cured acrylic resin, and it is often refractured at the repaired site. Self-cured acrylic resin is widely used as an acceptable repair material [2]. There have been innumerable attempts from researchers to modify the self-cured PMMA resin to enhance its mechanical performance.

A satisfactory repair must be able to maintain dimensional stability. Also, it needs to have adequate strength and pleasing aesthetics, on top of being a quick, simple, and relatively inexpensive procedure. To improve the physical and mechanical properties of acrylic resin, it was reinforced with various fibers and nanoparticles. The E-glass fiber has been extensively studied and is regarded as a form of PMMA reinforcement. However, the optimum concentrations for dental reinforcement and fibers sizes are inconclusive, and hence, it needs to be studied further. Using a short fiber-reinforced resin matrix, it appears to be a promising denture material to withstand high stress-bearing forces [3–5]. The sizing of glass fiber-reinforced composites with organofunctional silane assists the need of the fibers to transfer high stress across the fiber-matrix interphase, resulting in the ability to maintain the highest potential level of fiber strength and its capability to protect against physical, chemical, or environmental deterioration [6, 7]. Stipho concluded that denture acrylic resin reinforced with 1% glass fiber displayed the highest transverse strength of self-cured acrylic resin [1]. Yuliharsini et al. found that the addition of 1% chopped strands of 3 mm size E-glass fiber can improve the impact strength, transverse strength, and modulus of elasticity of heat-cured acrylic resin denture base [8]. Similar promising results were found in many studies of enhancing the positive effect of reinforcing acrylic denture base with E-glass fiber [9–11]. To achieve optimum properties, the interfacial adhesion between reinforcing filler particles with the matrix are crucial as it enables the transfer of load from the weak matrix to the reinforcing fillers [6, 7, 11–14]. As a result, it controls the whole mechanism of the crack propagation of the material. The reinforcement is typically based on microstructural factors, such as the type of resin used, manufacturing conditions, silanization of fibers [12, 13], particle percentage and distribution [1, 8, 10, 15], length and diameter [4, 16], fiber shape and orientation, [5, 16] along with the surface treatments of the particles [17, 18], which can affect the properties of the resin matrix.

In addition, the UHMW-PE filler is one of the most durable reinforcing fillers available, and as it is biocompatible and white in color, it is possible and preferable to use in aesthetic dental applications [4, 19]. Nevertheless, its inertness is the main drawback. The study by Gutteridge reported that the inclusion of 1% UHMW-PE fibers showed promising results for reinforcing acrylic resin, however, no significant effect was seen between untreated or plasma-etched UHMW-PE fibers [20]. Carlos concluded that the use of untreated UHMW-PE beads has no significant advantage on acrylic resin's properties [21]. Ranade et al. also concluded that UHMW-PE improved the toughness of the composites, however, it was with a decrease in flexural strength. In contrast, the study done by Alla et al. showed that concentrations as low as 1% UHMW-PE can improve the impact strength of denture base resin substantially [22].

The main goal of immense research efforts is to improve the performance of the dental acrylic resin and to increase the fracture toughness without decreasing other properties. The most common way to improve the fracture toughness of the brittle acrylic resin and enhance the UHMW-PE properties is through reinforcement with other filler particles. Carbon nanoparticles are a promising additive for enhancing the wear resistance and mechanical properties of UHMW-PE. Nanoalumina, nanosilica, nanotitanium dioxide, nanozirconium oxide [23, 24], silver, copper, alumina powder, talc, zeolite, nanoclay, aramid, hydroxyapatite (HA), polytetrafluorethylene (PTFE) [25], kevlar fiber, polyester fiber, and glass flakes [26] have also been reported to be able to enhance the properties of UHMW-PE [19]. There has also been an attempt to incorporate silver nanoparticles as an antimicrobial biomaterial without compromising but also enhancing the material's mechanical properties [27]. The versatility, affordability, and biocompatibility of glass fiber and polyethylene fillers promises to be an expanding platform for innovation by biomedical engineers and medical professionals in the coming future. Although reinforcing fillers and particles offers positive effects on the properties of the denture base acrylic resin [15], there are inadequate studies on the hybrid reinforcement of SEGF and UHMW-PE, and hence, more research is required.

Even though a vast variety of resin materials and techniques are available for processing dentures, the quest for the ideal acrylic resin material continues to be a focus of modern dental research. Therefore, this study was conducted to evaluate and compare the effects of SEGF and UHMW-PE filler particles addition on the flexural strength of self-cured PMMA denture base. The null hypothesis was that there were no statistically significant differences in the flexural strength of the self-cured acrylic resin incorporated with different percent by weight of SEGF and UHMW-PE filler particles.

## 2. Materials and Methods

### 2.1. Preparation of Reinforced Self-Cured Acrylic Resin

The self-cured acrylic resin employed in this study is Unifast Trad (Unifast™ Trad, self-curing acrylic resin, GC Corporation, Japan; Lot no. 2105141), and it is supplied in a powder-liquid form. A commercial discontinuous E-glass fiber (as-received silanized), with a diameter of 16 *μ*m and a length of 220 *μ*m, was obtained from Fibertec (Bridgewater, MA, USA; Lot no. NI022) and was used as received. The UHMW-PE filler particles (Ø150 *μ*m, PSD X50 Lot no. 200410018), received from IRPC (Bangkok, Thailand), were further surface-treated with the chromic acid solution [K_2_Cr_2_0_7_ : H_2_SO_4_ : H_2_O] of (7 : 150 : 12 wt%), following Li et al., before using it as a filler for reinforcing self-cured acrylic resin in this study [18, 28]. The SEGF and UHMW-PE filler particles were preweighted using an analytical balance (AND: GR 200, Japan). The reinforced self-cured acrylic resin was prepared by mixing the resin polymer powder with preweighed E-glass fiber and UHMW-PE filler particles according to each group percent by weight, as shown in Table 1, using a magnetic stirrer machine (IKA® RW20 digital, Staufen, Germany) at 450 rpm for 30 minutes, to achieve an equal and uniform distribution of particles in the resin polymer powder.

### 2.2. Preparation of Specimens for Flexural Strength Test

The stainless steel mold of dimension (64 × 10 × 3.3 mm^3^) was used to prepare the specimens for the flexural strength test (ISO standard 20795–1 : 2013). A total of fifty-six specimens were fabricated and were divided into seven groups—each with eight specimens. Separating media was applied onto the stainless-steel mold to prevent the adhesion of specimens to the mold. For each group, eight experimental specimens were fabricated by the simple mixing of reinforced self-cured acrylic resin powder with a liquid monomer, according to the manufacturer's recommendations. The procedure was started by measuring the liquid monomer in the rubber cup, followed by the prepared reinforced self-cured acrylic resin powder. Then, mix the powder and liquid together within 15 seconds of working time, and pour the mixture into the mold slightly in excess to compensate the polymerization shrinkage. The stainless-steel mold was screwed tightly from all angles to ensure that equal pressure was applied. After a setting time of 2 minutes and 30 seconds, the mold was placed in water at a temperature of 50°C for 5 minutes to ensure a complete polymerization reaction. All specimens were removed from the mold and polished with an automatic polishing machine that consists of abrasive silicon carbide 600, 800, 1000, and 1200-grit. All specimens were immersed into an ultrasonic machine for 1 minute for the cleaning and removal of dirty particles. Each specimen was standardized and inspected for any faults or voids and checked for the presence of any over edge materials. The thickness, width, and length of each specimen was examined for accuracy with a digital caliper (minimum reading 0.001 mm). All specimens were then stored in distilled water in an incubator at 37 ± 1°C for 50 ± 2 hours prior to the flexural strength test.

### 2.3. Test Method

For all fifty-six prepared specimens, the midpoint of each specimen was measured and marked. The flexural strength test was performed using a three-point bending testing device under a universal testing machine (EZ Test Series, Shimadzu, Japan). According to ISO 20795–1:2013, the device is set with a loading wedge and a pair of adjustable supporting wedges placed 50 mm apart. Force is applied in the center of the specimens with a load cell of 500 N and a crosshead speed of 5 mm/min. A uniformly increasing force was applied until the fracture of the specimens. The highest fracture load of each specimen was recorded and calculated in megapascals (MPa) using the following formula: *σ* = 3FL/2wd^2^, where F is the load (force) at the fracture point (N), *L* is the length of the support span (mm), *w* is the width (mm), and *d* is the thickness (mm).

### 2.4. Characterization of Reinforced Self-Cured Acrylic Resin

A scanning electron microscopy (SEM, JEOL, JSM- 6610LV, Oxford X-Max 50, Tokyo, Japan) was used to visualize the surface topography and examine the fillers' morphologies and distribution in the polymer matrix of the control and experimental specimens. After the specimens fractured, one sample of each group was randomly chosen. The fractured surfaces were gold-sputter-coated for 60 seconds at a sputter current of 25 mA of about 2 nm thickness before examining under the SEM. The microstructure analysis was performed at a voltage of 15 kV and 100x, 500x, and 1000x magnifications.

### 2.5. Statistical Analysis

The results of this study were analyzed using a statistical software (SPSS Statistics 22.0; SPSS Inc., IL, USA). The test of normality was performed using Kolmogorov-Smirnov's test, and the homogeneity of variances using Levene's test showed no violation of assumption. The parameter of one-way ANOVA was analyzed, followed by Tukey HSD multiple comparison post hoc test, to find means that are significantly different between each group of flexural strengths. Tests were at a confidence level of 95% and with a significant level of 0.05 (*α* = 0.05).

## 3. Results

**Figure 1:**
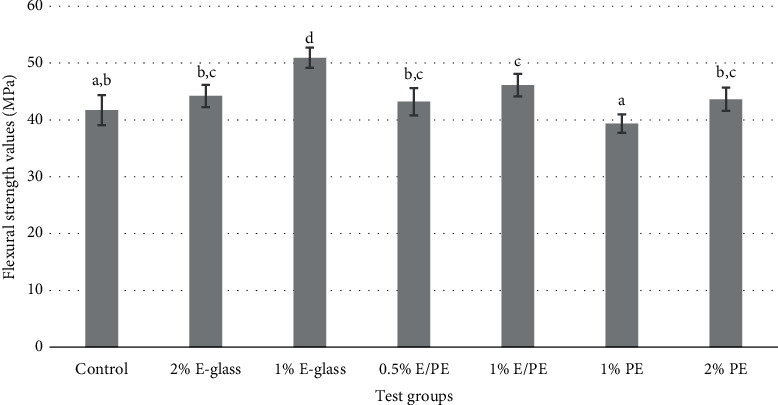
Bar chart representing the means and standard deviations of flexural strength test (MPa) of each tested group. Groups with the same lowercase superscripted letter indicated no significant differences between groups at *p* value<0.05.

The study was conducted to evaluate the effect of SEGF and UHMW-PE on the flexural strength of self-cured acrylic resin. The means, standard deviations, and statistical significance of flexural strengths (MPa) of the tested groups are summarized in Figure 1. By comparing all reinforced groups of self-cured acrylic resin, group 3 (1% E-glass) showed the highest flexural strength value, followed by group 5 (1% E/PE), group 2 (2% E-glass), group 7 (2% PE), group 4 (0.5% E/PE), and control group, while group 6 (1% PE) had the lowest flexural strength (50.93, 46.13, 44.22, 43.64, 43.21, 41.72, and 39.34, respectively).

As shown in Figure 1, the addition of SEGF and UHMW-PE significantly increased the flexural strength in group 1 (1% E-glass) and group 5 (1% E/PE) when compared to the control group (*p* < 0.05). The decrease in the flexural strengths of group 6 (1% PE) and the control group had no significant difference. The 1% PE group with the lowest flexural strength (39.34 ± 1.64 MPa) showed significant difference between all other reinforced groups: group 2 (2% E-glass), group 3 (1% E-glass), group 4 (0.5% E/PE), group 5 (1% E/PE), and group 7 (2% PE) (*p* < 0.001, 0.001, 0.01,0.001, respectively). The addition of 1% SEGF significantly increased the flexural strength (*p* < 0.001), and its increase was concentration dependent, where the highest flexural strength value was reported with 1% SEGF (50.93 MPa ± 1.78). A significant decrease in flexural strength was reported (*p* < 0.001) as the filler concentration increased to 2% SEGF (44.22 ± 1.95 MPa).

**Figure 2:**
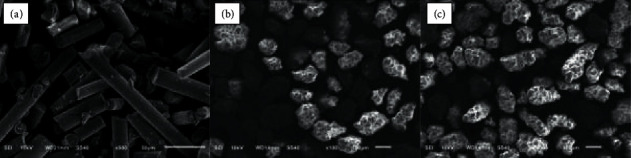
Representative SEM images (x100, x500 magnification) showing (a) short E-glass fiber, (b) ultra-high-molecular-weight polyethylene filler particles after surface modification, and (c) ultra-high-molecular-weight polyethylene filler particles before surface modification.

The microscopic characterization of reinforced particles using SEM as shown in Figure 2 revealed that SEGF are rod-shaped discontinuous short fibers that are randomly oriented. SEGF has a diameter of 16 *μ*m and an average length of 220 *μ*m (×500 magnification). On the other hand, UHMW-PE are much larger in size and are irregular in shape with various morphological surfaces of diameter that ranges from 80–150 *μ*m (×100 magnification). There were no prominent differences noted on the surface morphology after surface modification with the chromic acid solution.

**Figure 3:**
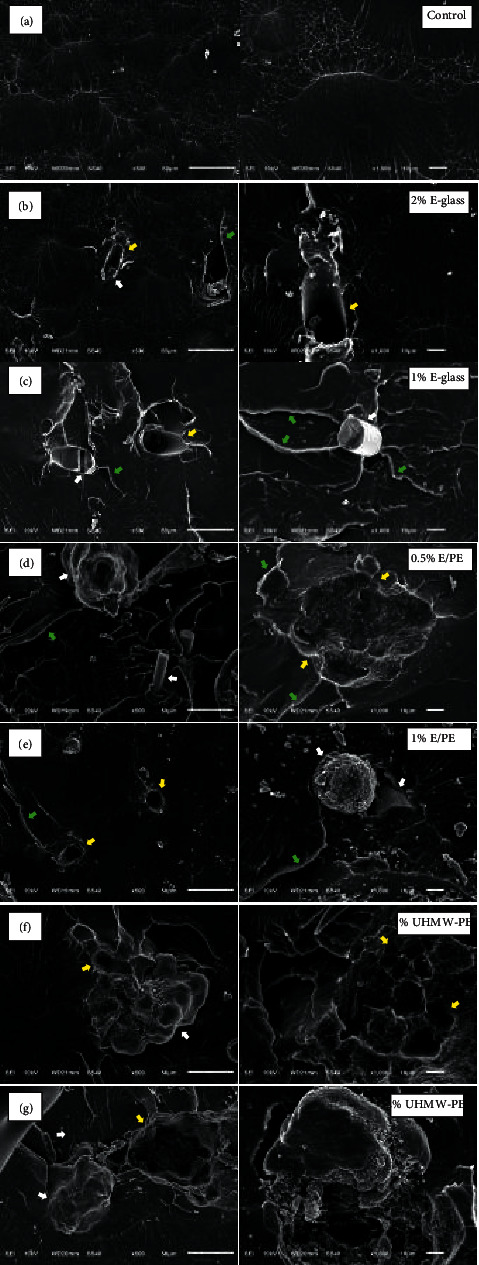
Representative SEM images (x500, x1000 magnification) showing the fractured surface of (a) self-cured acrylic resin (control group 1). (b) 2% short E-glass fiber reinforced self-cured acrylic resin (group 2). (c) 1% short E-glass fiber reinforced self-cured acrylic resin (group 3). (d) 0.5% short E-glass fiber/ultra-high-molecular-weight polyethylene filler-reinforced self-cured acrylic resin (group 4). (e) 1% short E-glass fiber/ultra-high-molecular-weight-polyethylene filler-reinforced self-cured acrylic resin (group 5). (f) 1% ultra-high-molecular-weight polyethylene filler-reinforced self-cured acrylic resin (group 6). (g) 2% ultra-high-molecular-weight polyethylene filler-reinforced self-cured acrylic resin (group 7). The white arrow indicates the reinforcement particles, the yellow arrow indicates the area that reinforced particle breakdown or that is dislodged, and the green arrow indicates the area of the cohesive failure of the resin.

The microscopic characterization with 500x and 1000x magnification of reinforced self-cured acrylic resin of all tested groups were taken from the fractured surface of the specimens (Figure 3). The control group with no addition of reinforced particles exhibited a smooth surface when compared to the other tested groups. A homogenous resin polymer matrix can be seen in the control group. The addition of reinforced particles to PMMA resin (groups 2–7) showed comparatively rougher surfaces with varying morphological features. The reinforced particles were widely and randomly distributed within the resin matrix. The presence of reinforced particles increased as the weight percentage of reinforcement increased. Fractures occurred between PMMA and PMMA (cohesive failure) within the material itself and at the interface of reinforced particles and PMMA (adhesive failure). In groups 2-3, with the addition of SEGF, glass fibers were noted to fail adhesively and protruded on the fracture surface with the formation of voids, as the fibers were pulled out to the opposing side of the fractured specimen. SEGF were seen to be partly or totally dislodged along with the breakage of the fiber itself (Figures 3(b) and 3(c)). Surface morphologies for groups 6-7, with the addition of UHMW-PE, were noted differently under SEM micrographs. Void formation, craters, and irregular morphological features were noted more where the particles detached. The majority of UHMW-PE particles were seen to breakdown into small pieces, not as a whole (Figures 3(f) and 3(g)). The clusters of particles were noted when the reinforcement concentration increased (Figure 3(g)). Both features of SEGF and UHMW-PE were seen in groups 4-5 (Figures 3(d) and 3(e)). Reinforced particle detachment during fracture presented an adhesive type of failure with hollow spaces between SEGF and UHMW-PE and the resin matrix. Crack propagation is also seen passing through the particles. Void formation was noted throughout the fracture surface of all tested groups.

## 4. Discussion

This study was conducted to evaluate and compare the effects of adding SEGF and UHMW-PE on the flexural strength of the repaired self-cured acrylic denture base to improve the denture base strength. The null hypothesis was rejected, where the addition of SEGF and UHMW-PE significantly affected the flexural strength of the reinforced self-cured acrylic resin. The results of this study demonstrated that the flexural strength of self-cured acrylic resin significantly increased after the addition of 1% SEGF +1% UHMW-PE and 1% SEGF. The results of this study are consistent with the results of prior investigations [8, 29, 30] that evaluated the effects of E-glass fibers and UHMW-PE, in which those studies also showed promising outcomes on the flexural strength of reinforcing PMMA dentures with E-glass. The reinforcement is affected by the volume or concentration added to the acrylic resin powder. Stipho concluded that the strength was the highest when the short-rod glass fiber was 1% of the polymer volume. The study also reported that the reinforcement of PMMA acrylic resin with low glass fiber concentrations was found to enhance the postrepair yield and the fracture strength of the resin. Incorporating more than 5% glass fiber content did not provide any substantial mechanical benefits [1]. Similarly, the study by Yuliharsini et al. found significant differences between heat-cured acrylic resin with and without the addition of 1% and 1.5% E-glass fiber [8]. There was a resemblance to the findings of the current study, where the addition of a higher fiber concentration of 2% SEGF shows no significant increase in flexural strength compared to the control group. It could be explained by the increase in the flexural strength with low concentration, which may be attributed to the homogenous distribution of particles and the particles' ability to fill interpolymeric chain spaces, while high concentrations may cause possible agglomeration, which forms spaces. The void spaces could explain the decrease in the strength of the material and the nonhomogeneous mixing [26]. These hollow spaces between the matrix discontinues the stress distribution, which led to weak points in the structure that eventually weakened the material. In addition, the resin matrix cannot absorb any further filler once the saturation point has been reached. The addition of excessive fillers disrupts the continuity of the resin matrix, reduces the bulk, and compromises the properties of the acrylic resin [31]. However, Nakamura et al. reported that using the nanoparticle size of PMMA powder reinforced with a higher content of short-rod glass fiber significantly improved the mechanical properties, and it was explained by the differences between PMMA powder size (150 *μ*m) and E-glass fiber (10 *μ*m), which led to difficulty in homogenizing the mixture and caused void space formation within the material [10]. Similarly, a study done by Alhotan et al. concluded that 3 to 7 percent by weight of E-glass fiber is an optimal filler concentration for reinforcing PMMA denture base resins [32]. It may be a result of the use of E-glass nanoparticles size as it can ensure the implantation of fillers in the resin and produce a homogenous mixture as it can also penetrate between the linear macromolecule chains, thereby limiting polymer chain movement [15, 24]. Solnit reported an increase in flexural strength with silanated short-rod glass fibers and reported a more homogenous mixture of PMMA and fiber [14]. Silane coupling agent, which helps to improve the chemical bond between the filler and the PMMA matrix, contributes to the greater amount of energy that is required to break the chemical bonds that form between the materials. The presilanized E-glass fiber could cause chemical bond formation between the fiber and the matrix. When the optimal level of the filler of the matrix is reached, superior fracture resistance was seen from the result in this study with the addition of 1% SEGF. Fiber reinforcement is also affected by fiber orientation. Vallittu suggested that the highest strength of fiber composite can be obtained with the fiber oriented in one direction, and the ultimate tensile strength of the material is reduced by changing continuous unidirectional fibers to longitudinally-oriented discontinuous short fibers of lower aspect ratio [16]. However, where stresses are multidirectional, discontinuous fibers are normally used. As seen under SEM in this study (Figures 3(b) and 3(c)), the failure types of discontinuous short fiber reinforced resin include the cracking of the polymer matrix, debonding and partial fracturing of SEGF, or totally being pulled out from the resin matrix.

The results of this study also demonstrated a decrease in the flexural strength of the self-cured acrylic resin after the addition of 1% UHMW-PE and slightly increased with 2% UHMW-PE. However, this weakening of the resin was not statistically significant when compared to the control group. A similar result was seen from the study by Gutteridge that the inclusion of 1% untreated UHMW-PE fiber may reduce the transverse strength of acrylic resin but increased with the addition of 2% untreated UHMW-PE fiber [20]. The decreased in flexural strength with addition of 1% UHMW-PE filler may be a result from the insufficient concentration added to incur any beneficial enhancement effect of acrylic resin but in turn rather be a weak point of the resin. In addition, it could also be attributed to the heterogeneous mixture and that the presence of filler particles reduces the bulk of the acrylic resin leading to decreased in flexural strength. On the other hand, the increase in flexural strength with 2% UHMW-PE filler may be a result of the effectiveness of the surface treatments and the irregularity of the particles' morphology, which might result in the micromechanical interaction between the resin and the matrix. This confirmed the idea that nonbonded interactions are the main source of interaction between the resin and the UHMW-PE filler. The UHMW-PE did leave craters impressions after dislodging from the fractured surfaces. The craters were about the same size as the original filler's average particle size. SEM images (Figures 3(f) and 3(g)) can provide insights into this toughening effect of UHMW-PE, where fractures were seen to have occurred through the particles themselves (Figure 3(g)). The propagation of the crack through the UHMW-PE particles yields small fibrils, and it leads to the formation of microvoids and small crack lines around the reinforced particles. In addition, the particles' agglomeration was also noted (Figure 3(g)), which acted as the stress concentration area caused by the weakening of the resin matrix interphase, and therefore, it decreased the flexural strength of self-cured acrylic resin compared to other reinforced groups [20]. A conjunction with the study by Uzun Hersek reported that the lowest transverse strength was obtained with polyethylene fibers, which also insignificantly decreased the transverse strength of the acrylic resin [33]. Dixon and Breeding reported that no significant increase of transverse strength was seen with different acrylic resins reinforced with the polyethylene fiber [34]. Carlos Harrison concluded that the use of untreated UHMW-PE beads has no significant benefit on the acrylic resin's properties [21]. Ranade et al. also concluded that the addition of UHMW-PE improved the toughness and modulus of the composites, however, with a decrease in flexural strength [35]. In contrast to the short-rod E-glass fiber, it is advantageous over large molecules of UHMW-PE, which can be investigated under SEM. The SEM examination (Figures 3(f) and 3(g)) of the fractured surface of the specimens revealed the irregular shape with the rough surfaces of UHMW-PE particles with average dimensions of 80–100 *μ*m *X* 100–150 *μ*m. The UHMW-PE particles were noted to be in many forms, among which the fractured, whole, still embedded in the matrix, and craters left by the particles being pulled out from the matrix were noted. In the SEM images shown in Figures 3(b) and 3(c), no difference in surface morphology was noted after the surface treatment of the UHMW-PE filler with the chromic acid solution. In contrast to this study, the study done by Li et al. showed that potassium dichromate treatment effectively changed the surface properties of UHMW-PE fibers and significantly improved the impact strength but lowered the tensile strength of epoxy resin [18].

Based on the SEM analysis of the fractured surface of specimens, the random and wide distribution of reinforced particles within the resin matrix and the presence of particles increased as the weight percentage of reinforcement increased, which could confirm the reliability of the magnetic stirrer and that it was an acceptable technique in mixing the PMMA powder and the reinforcements. This technique reduced human errors and aided in achieving a uniform distribution of particles throughout the specimens [32]. The appearance of particles clustering, which form spaces within the resin matrix, can be observed with the addition of the higher ratios of particles, especially UHMW-PE particles, supported by the SEM image (Figure 3(g)) group 7). The SEM image also revealed the presence of large porous structures, which could be a result of the agglomeration particles as shown by Nejatian et al., which causes the discontinuity of stress distribution, leading to weak points in the structure, and it causes a decrease in the mechanical performance of the reinforced PMMA resin [26]. Microscopic examination revealed partial bonding between the glass fibers and the resin matrix. The E-glass fibers failed adhesively and protruded with the formation voids because of the fibers being pulled out. It could indicate that the chemical bond formation between the fiber and the matrix was because of the effect of the presilanized treatment of the fiber. Therefore, when the optimal level of filler in the matrix is reached, the superior fracture resistance of the 1% E-glass reinforced group can be marked compared to the other tested groups. In cases where the levels of effective bonding between the reinforced particles and the matrix are achieved during flexural loading, the reinforced particles should act as stress-bearing areas, and equal stress distribution may have been established, which improves flexural strength and resistance to reinforced particles fracture.

One of the main limitations of the study was that there was just one commercial acrylic resin used and one mechanical test performed. Thus, different findings might be obtained from different types of denture bases, and other mechanical properties should be investigated. Among alternative fillers for dental applications, short E-glass fibers may provide the best aesthetic qualities and prove to enhance the flexural strength of the PMMA denture base. The cytotoxicity and biological effects of the reinforced materials, as well as whether reinforced self-cured denture base resin in the mouth may draw more plaque or induce gingival irritation, are yet to be investigated.

## 5. Conclusions

The self-cured PMMA resin will remain to be the preferred material of choice for the reparation of denture prostheses. Short E-glass fiber showed significant improvement in the flexural strength of the materials. Clinically, this technique of the simple addition of E-glass fiber to self-cured PMMA resin for repairing fractured dentures could work as a quick method and easy solution with cost-effectiveness and faster turnover of lab work for the patient in improving the strength of the self-cured PMMA resin. Within the constraints of this research study, it can be concluded that the addition of 1% SEGF to self-cured PMMA acrylic resin significantly improved the flexural strength.

## Figures and Tables

**Table 1:** Specimen grouping according to acrylic powder, short E-glass fiber, and ultra-high-molecular-weight polyethylene filler percentage.

Group	Reinforcement + PMMA acrylic powder (%wt)	
1	Control	(0% SEGF + 0% UHMW-PE) + 100% PMMA acrylic powder
2	2% E-glass	(2% SEGF + 0% UHMW-PE) + 98% PMMA acrylic powder
3	1% E-glass	(1% SEGF + 0% UHMW-PE) + 99% PMMA acrylic powder
4	0.5% e/pe	(0.5% SEGF + 0.5% UHMW-PE) + 99% PMMA acrylic powder
5	1% e/pe	(1% SEGF + 1% UHMW-PE) + 98% PMMA acrylic powder
6	1% PE	(0% SEGF + 1% UHMW-PE) + 99% PMMA acrylic powder
7	2% PE	(0% SEGF + 2% UHMW-PE) + 98% PMMA acrylic powder

## Data Availability

The data used to support the findings of this study are included within the article and are also be available from the corresponding author upon request.
